# Gne-Depletion in C2C12 Myoblasts Leads to Alterations in Glycosylation and Myopathogene Expression

**DOI:** 10.3390/cells15020199

**Published:** 2026-01-20

**Authors:** Carolin T. Neu, Aristotelis Antonopoulos, Anne Dell, Stuart M. Haslam, Rüdiger Horstkorte

**Affiliations:** 1 Institute for Physiological Chemistry, Medical Faculty, Martin-Luther-University Halle-Wittenberg, 06114 Halle, Germany; 2 Department of Life Sciences, Imperial College London, London SW7 2AZ, UKs.haslam@imperial.ac.uk (S.M.H.)

**Keywords:** GNE, GNE-myopathy, myopathogenes, sialic acids, glycosylation, N-glycans

## Abstract

GNE myopathy is a rare genetic neuromuscular disorder caused by mutations in the *GNE* gene. The respective gene product, UDP-*N*-acetylglucosamine 2-epimerase/*N*-acetylmannosamine kinase (GNE), is a bifunctional enzyme that initiates endogenous sialic acid biosynthesis. Sialic acids are important building blocks for the glycosylation machinery of cells and are typically found at the terminal ends of glycoprotein N- and O-glycans. The exact pathomechanism of GNE myopathy remains elusive, and a better understanding of the disease is urgently needed for the development of therapeutic strategies. The purpose of this study was to examine the effects of hyposialylation on glycan structures and subsequent downstream effects in the C2C12 Gne knockout cell model. No overall remodeling of N-glycans was observed in the absence of Gne, but differences in glycosaminoglycan expression and *O*-GlcNAcylation were detected. Expression analysis of myopathogenes revealed concomitant down-regulation of muscle-specific genes. Among the top candidates were the sodium channel protein type 4 subunit α (*Scn4a*), voltage-dependent L-type calcium channel subunit α-1s (*Cacna1s*), ryanodine receptor 1 (*Ryr1*), and glycogen phosphorylase (*Pygm*), which are associated with excitation-contraction coupling and energy metabolism. The results suggest that remodeling of the glycome could have detrimental effects on intracellular signaling, excitability of skeletal muscle tissue, and glucose metabolism.

## 1. Introduction

Glycosylation is a versatile and highly abundant post-translational modification, which consists of the attachment of glycans to protein and lipid backbones. Nine different monosaccharides compose the main building blocks for mammalian glycans, sialic acids being one of them. Sialylation always occurs on the terminal ends of glycans and serves a variety of different physiological functions. The bifunctional enzyme UDP-*N*-acetylglucosamine 2-epimerase/*N*-acetylmannosamine kinase (GNE) plays a key role in initiating endogenous sialic acid biosynthesis, and mutations in GNE are known to cause the rare neuromuscular disorder GNE myopathy (GNEM) [1,2]. GNEM is characterized by an early adulthood-onset and slowly progressive distal and proximal muscle weakness [3]. As there is no therapy available, most patients become wheelchair-bound within 10–20 years after disease onset [4]. After decades of GNEM research, the pathomechanism remains elusive, but due to the role of GNE in sialic acid biosynthesis, it is commonly assumed that hyposialylation of skeletal muscle tissue is the main driver of the disease [5]. However, other studies could not find a global reduction in sialylation, even by concomitant impaired GNE-epimerase activity [6,7].

Even though GNE is ubiquitously expressed and sialylation is important throughout the body, GNE variants only affect the skeletal muscle tissue. Skeletal muscle is an excitable tissue and has to withstand major mechanical stresses upon contraction and relaxation cycles, requiring a special architecture of the myofiber membranes (sarcolemma). Furthermore, its regenerative capacity relies on the maintenance of a quiescent satellite cell pool that can be activated upon injury for differentiation and replacement of damaged fibers. Interestingly, Gne protein and mRNA levels were found to be up-regulated in response to muscle injury in a mouse model, suggesting the important role of sialic acids during muscle regeneration [8]. This finding is supported by several studies that found that loss of functional Gne impaired myoblast differentiation [9,10,11]. Furthermore, glycosylation, in general, is dynamically regulated during myoblast differentiation [12]. A glycoproteomic study validated the remodeling of N-glycans during myogenesis and demonstrated alterations in galectin-1 binding, therefore linking it directly to muscle development in a mouse model [13]. However, investigations of satellite cell-opathies, a group of muscle disorders that are directly linked to satellite cell function, identified GNE as a Pax7-regulated myopathogene that is down-regulated in the first 5 h of satellite cell activation [14,15]. Dynamic regulation of GNE expression would be complementary to the findings of Chen and co-workers, who proposed a temporal decrease in sialic acids during differentiation of C2C12 myoblasts [16]. Thus, it is likely that sialic acids serve different roles during myogenesis and in mature myofibers, respectively, and both should be considered in terms of the pathophysiology of GNEM.

The aim of this study was to elucidate the (patho-)physiological consequences of the manipulation of the sialic acid pathway in skeletal muscle cells. We used C2C12 Gne knockout and Sol8 Gne knockout cell lines as models to investigate alterations in the glycosylation machinery of muscle cells that are hyposialylated. Furthermore, we identified several myopathogenes that are down-regulated upon Gne knockout. Among those genes are the sodium channel protein type 4 subunit α (*Scn4a*) and voltage-dependent L-type calcium channel subunit α-1s (*Cacna1s*), both of which are important for skeletal muscle physiology and excitation-contraction coupling.

## 2. Materials and Methods

Cell culture and myoblast differentiation. C2C12 and Sol8 cells were cultured in DMEM (Dulbecco’s Modified Eagle’s Medium; 11960044; Gibco/Thermo Fisher Scientific; Waltham, MA, USA) supplemented with 10% FBS (Fetal Bovine Serum; A5256801; Gibco/Thermo Fisher Scientific; Waltham, MA, USA) and 1% L-glutamine (L-Gln; 200 mM; A2916801; Gibco/Thermo Fisher Scientific; Waltham, MA, USA) at 37 °C in a humidified atmosphere with 5% CO_2_. For C2C12 and Sol8 differentiation, dishes were coated with 0.1% gelatine 24 h prior to cell seeding, and normal growth medium (GM) was replaced by differentiation medium (DM). DM consisted of DMEM supplemented with 2% FBS and 1% L-Gln. C2C12 cells (ACC 565) were purchased from DSMZ (Braunschweig, Germany). C2C12 Gne knockout clones #24 and #26 were generated as previously described [11]. Sol8 wild-type (ATCC CRL-2174) and Sol8 Gne knockout cells were kindly provided by Stella Mitrani-Rosenbaum (Hadassah—The Hebrew University Medical Center, Jerusalem, Israel) [10]. N-acetylneuraminic acid (Neu5Ac) was purchased from Molekula Group GmbH (39596039-10g; Munich, Germany).

Western blot./*Immunoblot.* Western Blots were performed as described earlier [11]. Cells were washed twice with ice-cold PBS and harvested from the culture dish using a cell scraper to avoid membrane protein modifications. RIPA buffer (25 mM Tris-HCl pH 7.5, 150 mM NaCl, 1% NP-40, 1% sodium deoxycholate, 0.1% SDS) containing 1× protease inhibitor cocktail (Sigma Aldrich; St. Louis, MO, USA) and 1 mM PMSF was used for cell lysis. Lysates were incubated on ice for 30 min and total protein was isolated by centrifugation at 14,000× *g* at 4 °C for 10 min and quantified using the Pierce™ BCA Protein Assay Kit (23225; Thermo Fisher Scientific; Waltham, MA, USA). Equal amounts of protein were mixed with 5× SDS-laemmli buffer (containing 50 mM DTT) and separated on an 8–10% gel. Proteins were transferred on a nitrocellulose membrane and stained with Ponceau S for normalization. Membranes were blocked with 5% skimmed milk in TBS-Tween (TBS-T) for 1 h at room temperature prior to incubation with primary antibody overnight at 4 °C with agitation. On the next day, membranes were washed three times with TBS-T and subsequently incubated with secondary antibody for 1 h at room temperature (Goat anti-mouse IgG H&L HRP, 1:10,000, ab6789; abcam; Cambridge, UK). After three repeated washing steps with TBS-T, Immobilon^®^ Forte Western HRP Substrate was used for detection (Merck, Darmstadt, Germany) using the ChemiDoc MP imaging system from Bio-Rad Laboratories (Hercules, CA, USA). Signal quantification was performed using ImageJ Software (version 1.54r).

Immunofluorescence Staining (IF). Cells were grown on coverslips coated with 0.1% gelatine in a 12-well plate. After aspiration of the culture medium, cells were washed twice with pre-warmed PBS and fixed with pre-warmed 4% PFA for 10 min at room temperature. Following two washing steps with PBS, cells were permeabilized with 0.5% Triton X-100/PBS for 10 min at room temperature, washed two times with PBS, and blocked with 1% BSA/PBS for 30 min. The primary antibody was diluted 1:100 in 1% BSA/PBS and incubated overnight at 4 °C. Cells that served as negative control (secondary antibody only) were incubated with 1% BSA/PBS only. The next day, cover slips were washed again three times with PBS and incubated with Goat anti-Mouse IgG (H+L) Alexa Fluor^TM^ Plus 647 (Thermo Fisher Scientific; Waltham, MA, USA) and DAPI diluted 1:1000 in PBS for 1.5–2 h at room temperature, protected from light. Excess staining solution was removed by another three PBS washing steps, and coverslips were mounted on glass slides with mowiol mounting medium.

Primary antibodies and lectins used in this study are listed in Table 1:

Quantitative reverse transcription PCR (qRT-PCR). qPCR was perfomerd as described earlier [11]. Cell pellets from a 6-well plate were lysed in 1 mL Trizol reagent and transferred to a 1.5 mL tube. Total RNA was isolated by phenol/chloroform extraction. Briefly, samples were mixed with 200 µL chloroform, shaken vigorously and incubated at room temperature for 3 min. For phase separation, the samples were centrifuged for 15 min at 12,000× *g* at 4 °C. The aqueous phase containing the RNA was transferred to a new tube with 500 µL of isopropanol, mixed, and incubated at room temperature for 10 min. The samples were centrifuged for 20 min at 12,000× *g* at 4 °C to precipitate the RNA. The RNA pellet was washed with 1 mL of 70% ethanol and centrifuged at 8000× *g* at 4 °C for 5 min. The ethanol was removed, and the pellet was air-dried for 10 min before being resuspended in 20–50 µL RNase-free H_2_O. RNA concentration was determined using the DS-11 FX+ spectrophotometer (DeNovix; Wilmington, DE, USA). cDNA was synthesized, using 2 µg of total RNA and SuperScript™ II reverse transcriptase (18064022; 2000 units; Thermo Fisher Scientific; Waltham, MA, USA), following the manufacturer’s instructions. RT-qPCR was performed using qPCR SybrMaster (PCR-372S; Jena Bioscience; Jena, Germany) and the CFX Connect™ Real-Time PCR Detection System (1855201; Bio-Rad; Hercules, CA, USA). Cq values were normalized to the housekeeping gene Gapdh, and relative gene expression was calculated using the ΔCq-method. Primer sequences used for qPCR analysis are listed in Table 2.

Glycomic analysis of N-glycans. For N-glycan structural analysis, all cells were treated as described previously [17]. Briefly, cell pellets (harvested from two 15 cm plates) were subjected to sonication in lysis buffer in the presence of detergent (CHAPS). Subsequently, proteins were reduced in 4 M guanidine-HCl, carboxymethylated, and digested with trypsin. The digested glycoproteins were purified by C18-Sep-Pak (Waters Corp., Hertfordshire, UK). N-glycans were released by peptide N-glycosidase F digestion. Structures of released N-glycans were analysed by matrix-assisted laser desorption ionization-time of flight MS (MALDI-TOF MS) and MALDI-TOF/TOF MS/MS using a 4800 MALDI-TOF/TOF (Applied Biosystems) mass spectrometer. For data acquisition, permethylated samples were dissolved in 10 µL of methanol. 1 μL of dissolved sample was premixed with 1 μL of matrix (20 mg/mL 3,4-diaminobenzophenone in 75% (*v*/*v*) aqueous MeCN) before being spotted onto a target plate and dried under vacuum. MS data were processed using Data Explorer 4.9 Software (Applied Biosystems). The processed spectra were subjected to manual assignment and annotation with the aid of a glycobioinformatics tool, GlycoWorkBench [18]. The proposed assignments for the selected peaks were based on ^12^C isotopic composition according to the knowledge of the biosynthetic pathways of N-glycans in murine cells. Proposed structures were confirmed by data obtained from MS/MS experiments. Two independent glycan extractions from cells were performed and shown as representative results.

N-Glycan branching (PHA-L Lectin staining). 100,000 cells were seeded in a 6-well plate and cultivated for 48 h prior to lectin staining. Cells were washed with PBS and detached using 2× trypsin/EDTA solution for 3 min. Cells were resuspended in fresh medium and pelleted by centrifugation for 3 min at 500× *g* at 4 °C. For removal of medium remnants, cells were washed again with PBS. The cell pellets were resuspended in 100 µL staining solution (20 µg/mL PHA-L in PBS) and incubated for 1 h at 4 °C. Excess staining solution was removed by an additional three washing steps with PBS before cells were resuspended in 300 µL PBS for flow cytometry analysis. PHA-L positive cells were detected in the FL-1 channel using the 488 nm excitation laser. Unstained cells served as a negative control.

Periodic acid/Schiff’s reaction and Alcian blue staining. Cells were differentiated in a 12-well plate, coated with 0.1% gelatine, for 7 days. Cells were washed twice with pre-warmed PBS and fixed with 4% paraformaldehyde for 10 min at room temperature. After washing again with PBS, cells were incubated for 15 min in a filtered alcian blue (Sigma-Aldrich, A5268) staining solution (1% *w*/*v* alcian blue in 3% acetic acid), followed by 5 min incubation with 1% periodic acid. Cells were stained with Schiff’s reagent for 15 min, followed by counterstaining with hematoxylin solution for 2 min. All steps were performed at room temperature, and cells were rinsed with tap water after each step.

ATP-Glo^®^ Assay. To quantify intracellular ATP levels, the CellTiter-Glo^®^ 2.0 assay was used according to the manufacturer’s instructions (Promega; Fichtburg, WI, USA, G9241). Briefly, 8000 cells were seeded in a 96-well plate and cultured for either 48 h (myoblasts) or differentiated for 7 days (myotubes). ATP-Glo reagent was added directly into the wells in a 1:1 ratio with culture medium. Cells were lysed for two min on an orbital shaker and incubated for 10 min at room temperature before measuring the luminescence signal in a plate reader (CLARIOStarPlus, BMG Labtech, Ortenberg, Germany).

Statistical Analysis. Data analyses were performed using OriginPro 2019 software, and differences were considered significant when *p* < 0.05. Unpaired Student’s t-test and one-way ANOVA were used as appropriate. Visualization of graphs was finalized with CorelDRAW 2021. Figures show the average mean ± standard deviation. Statistical significance was indicated by * *p* < 0.05, ** *p* < 0.01, *** *p* < 0.001.

## 3. Results

### 3.1. Gne Depletion Causes Morphological Differences in C2C12 Myotubes

**Figure 1:**
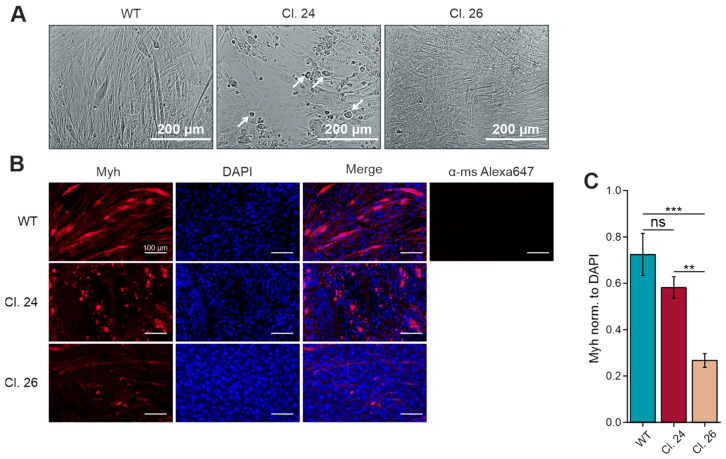
Differentiation of C2C12 wild-type and Gne knockout clones. (**A**) Micrographs of differentiated C2C12 wild-type (WT) and Gne^KO^ clone #24 and clone #26. (**B**) Immunofluorescent staining of Myh on day 7 of differentiation in C2C12 WT and Gne^KO^ clones. (**C**) Quantification of the Myh signal norm to DAPI. Myh: myosin heavy chain I. α-ms Alexa647: negative control (secondary antibody only). Micrographs and immunofluorescent staining show representative pictures. Scale bar in (**A**) 200 µm, in (**B**) 100 µm. The bar graph in (**C**) shows the mean of three independent micrographs ± standard deviation. Asterisks indicate the *p*-value of the sample compared to the wild-type control. Statistical analysis: One-way ANOVA. ns = non-significant, ** *p* < 0.01, *** *p* < 0.001.

We previously reported the impaired differentiation and myotube formation in C2C12 Gne knockout (Gne^KO^) clones compared to wild-type (WT) cells [11]. Addition of 10 nm insulin into the differentiation medium and coating of plates with 0.1% gelatine helped to restore myoblast fusion and differentiation to some extent (Figure 1). Nevertheless, immunofluorescence staining revealed that myosin heavy chain (Myh) expression was reduced and showed abnormal localization in the form of accumulation in Gne^KO^ clones (Figure 1B,C). Especially clone #24 showed characteristic alterations in cell morphology by forming extremely large myotubes and cell debris-like deposits (Figure 1A, white arrows).

### 3.2. Structural Analysis of N-Glycans in C2C12 Gne^KO^ Myoblasts

**Figure 2:**
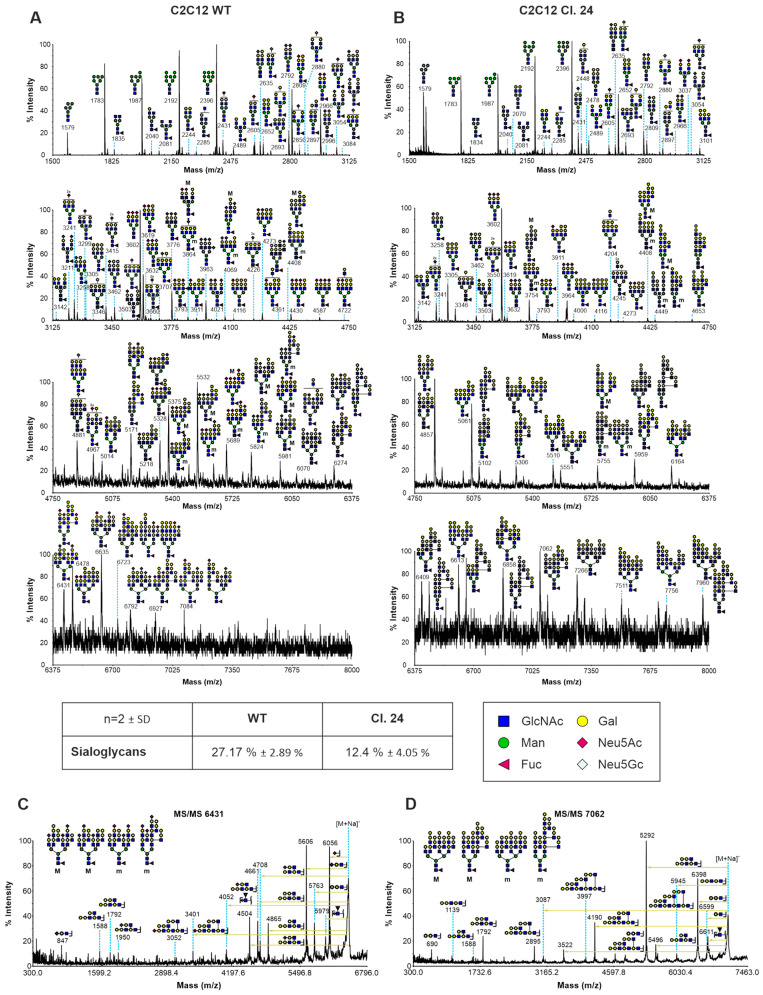
N-glycan structures of C2C12 wild-type and Gne^KO^ myoblasts. MALDI-TOF-TOF MS spectra of N-glycans obtained from panel (**A**) C2C12 wild-type myoblasts and panel (**B**) Gne^KO^ clone #24. Putative cartoon structures assigned were based on composition, tandem mass spectrometry, the literature, and knowledge of N-glycan biosynthetic pathways. All spectra were graphed as % relative intensity. All molecular ions are [M + Na]^+^. Experiments were repeated on two biological replicates, and the spectra shown are representative. (**C**) MS/MS spectrum of C2C12 wild-type m/z 6431 glycan. (**D**) MS/MS spectrum of C2C12 Gne^KO^ clone #24 m/z 7062 glycan. Isotopic glycan structures with “M” and “m” indicating major (M) and minor (m) abundancies, respectively. Quantification of sialoglycans in two independent experiments indicates that 27.17 ± 2.89% of the detected N-glycans are sialylated in wild-type cells, while there are 12.4 ± 4.05% sialoglycans in Gne^KO^ cells. GlcNAc: N-acetylglucosamine, Man: Mannose, Fuc: Fucose, Gal: Galactose, Neu5Ac: N-acetylneuraminic acid, Neu5Gc: N-glycolylneuraminic acid.

Sialylation has been previously shown to play a crucial role during muscle cell differentiation along with dynamic remodeling of sialoglycans [9,10,13,16]. However, it has not been investigated whether blockage of the endogenous sialic acid biosynthesis pathway by Gne depletion has other effects on N-glycan structures besides altering levels of sialic acids. Therefore, N-glycan analysis of C2C12 wild-type and Gne^KO^ clone #24 myoblasts was performed. Figure 2 shows that terminal sialic acids were mainly replaced by additional galactose residues, resulting in an increase in the galactose-α-1,3-galactose (α-Gal) epitopes in Gne^KO^ clone #24. The overall sialoglycan content was decreased by about 46%. Generally, both cell lines contained high mannose glycans, some bisected glycans, and complex N-glycans (Figure 2A,B). Higher mass glycans also contained chains of repeating units of galactose and N-acetylglucosamine (polyLacNAc). MS/MS analysis of selected high-mass glycans validated branched LacNAc (I-branches) structures (Figure 2C,D). However, global remodeling of the N-glycan core structures was not observed.

### 3.3. Fate of UDP-N-Acetylglucosamine in Gne Knockout Cells

**Figure 3:**
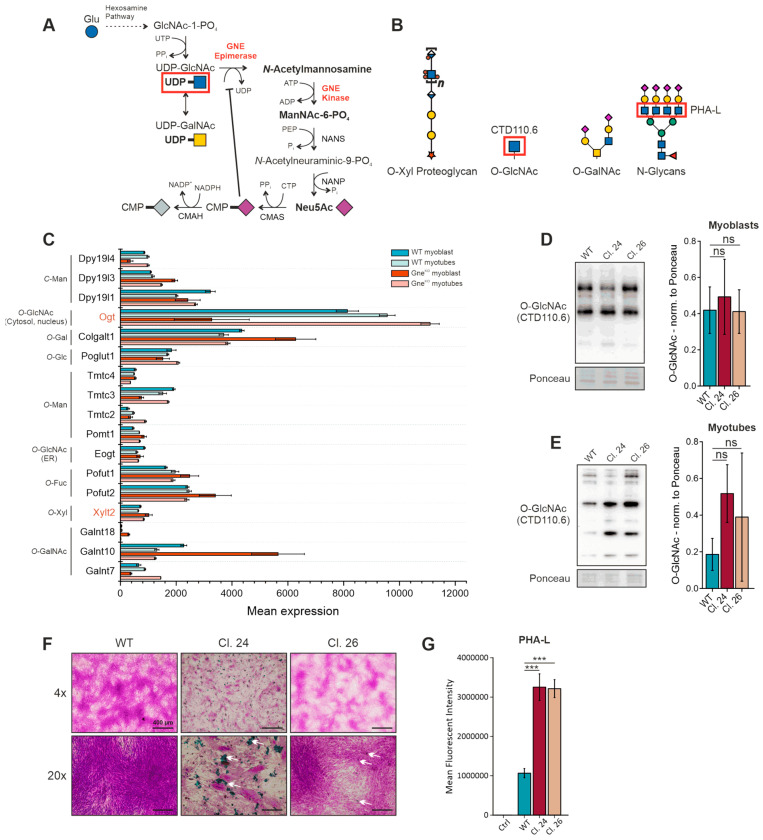
Analysis of glycosylation events in Gne^KO^ clones. (**A**) Endogenous sialic acid (*N*-acetylneuraminic acid) biosynthesis pathway. GNE catalyzes the epimerization of UDP-GlcNAc to ManNAc and its subsequent phosphorylation to ManNAc-6-PO_4_. (**B**) Common glycan structures that contain *N*-acetylglucosamine (GlcNAc). (**C**) Analysis of glycosylation-initiating gene expression in a Sol8 RNAseq data set [10]. (**D**) O-GlcNAcylation in C2C12 myoblasts. (**E**) O-GlcNAcylation in C2C12 myotubes. (**F**) Alcian blue/PAS staining of C2C12 myotubes. (**G**) N-glycan branching via PHA-L staining. GlcNAc: *N*-acetylglucosamine, GalNAc: *N*-acetylgalactosamine, PHA-L: phytohaemagglutinin-L, LacNAc: Galβ1-4GlcNAc, LacdiNAc: GalNAcβ1-4GlcNAc. Bar graphs show the mean of three independent experiments ± standard deviation. Asterisks indicate the *p*-value of the sample compared to the wild-type control. Statistical analysis: One-way ANOVA. ns = non-significant, *** *p* < 0.001.

Gne catalyzes the reaction of UDP-GlcNAc to ManNAc-6-PO_4_, directing the metabolic flux of UDP-GlcNAc to the biosynthesis of sialic acids (Figure 3A). In case of Gne depletion, the substrate UDP-GlcNAc is prone to accumulation and hence, has to follow different metabolic pathways. UDP-GlcNAc itself is used as a donor for glycan synthesis and is found in *O*-xylose proteoglycans, in *O*-GalNAc structures, in N-glycans, and in intracellular *O*-GlcNAcylation (Figure 3B). The transcriptomic data set of the Sol8 skeletal muscle Gne knockout cell line [10] was used to analyze changes in the expression of glycosylation-initiating genes (Figure 3C). The data suggest disregulation of enzymes that are involved in O-glycosylation and C-mannosylation. Despite many interesting candidates, we first followed up on UDP-*N*-acetylglucosamine-peptide *N*-acetylglucosaminyltransferase (Ogt)-mediated *O*-GlcNAcylation, as Ogt was down-regulated in Sol8 Gne^KO^ myoblasts and up-regulated in Gne^KO^ myotubes. Ogt transfers a single UDP-GlcNAc to a serine or threonine residue of cytoplasmic or nuclear proteins, serving as a master switch to dynamically regulate protein and transcription factor activities [19]. While there were no marked differences in *O*-GlcNAc levels in C2C12 Gne^KO^ myoblasts compared to wild-type cells, myotubes showed a strong tendency to increased *O*-GlcNAcylation (Figure 3D,E). The large error bars in the Western blot quantification data highlight the dynamic nature of this post-translational modification. Nevertheless, *O*-GlcNAcylation was shown to have negative effects on glucose uptake and contractile functions of myofilaments; thus, even slight changes could have pathophysiological consequences in GNEM subjects [20,21]. Additionally, xylosyltransferase 2 (Xylt2) was among the candidates that were up-regulated in Gne^KO^ myoblasts as well as in myotubes. Xylt2 initiates proteoglycan synthesis; thus, alcian blue staining in combination with the periodic acid/Schiff’s (PAS) reaction was used to visualize sulfated glycosaminoglycans and glycoproteins, respectively. C2C12 Gne^KO^ myotubes showed reduced PAS reactivity with concomitant increased abundance of alcian blue positive material in the extracellular milieu (Figure 3F, white arrows). Lastly, N-glycan branching of myoblasts was analyzed via PHA-L lectin staining and flow cytometry, where both Gne^KO^ clones showed a strong increase in PHA-L reactivity (Figure 3G). In summary, the different approaches to identify changes in GlcNAc-related glycan structures indicated alterations in the extracellular matrix composition and in intracellular signaling cues.

### 3.4. De-Regulation of Myopathy-Related Genes in Gne^KO^ Cells

**Figure 4:**
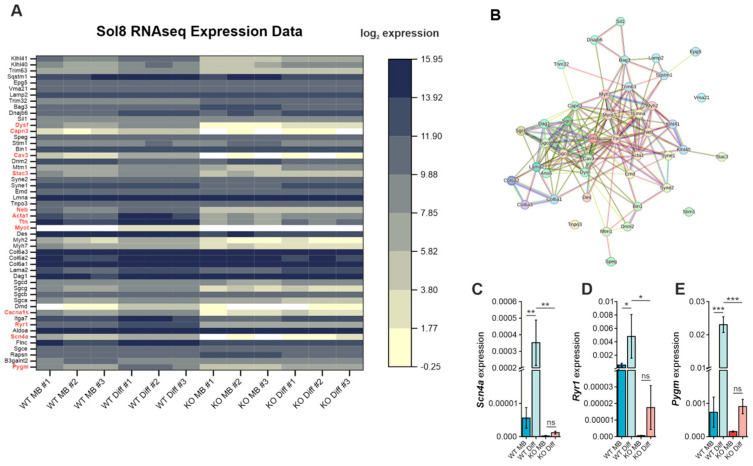
Heatmap showing differential gene expression of myopathogenes in Sol8 wild-type (WT) and Gne^KO^ (KO) cells. (**A**) Computational analysis of a manually selected subset of genes from a published data set of Sol8 wild-type and Gne^KO^ RNA-seq analysis. Gene expression is plotted as log2 RPKM. Genes of special interest are highlighted in red. WT: Sol8 wild-type, KO: Sol8 Gne^KO^, MB: myoblast, Diff: differentiated. Each column shows the expression of one biological experiment (#1–#3). Expression data were manually drawn from the data set published in [10]. (**B**) Interaction network of the gene products that are listed in the heatmap according to the STRING database. (**C**) mRNA expression of the sodium channel protein type 4 subunit α (Scn4a). (**D**) mRNA expression of the ryanodine receptor 1 (Ryr1). (**E**) mRNA expression of the muscle-specific glycogen phosphorylase (Pygm). All values represent the ΔCt value norm. to Gapdh expression. MB: myoblast, Diff: differentiated. Bar graphs show the mean of three independent experiments ± standard deviation. Statistical analysis: One-way ANOVA. ns = non-significant, * *p* < 0.05, ** *p* < 0.01, *** *p* < 0.001.

Next, we sought to investigate the functional effects of aberrant glycosylation in Gne^KO^ cells. As GNE myopathy solely affects skeletal muscle tissue, we wanted to focus on myopathy-related genes that have important functions in the maintenance of proper muscle physiology. The transcriptomic data set of the Sol8 Gne^KO^ cells served again as a starting point for the identification of candidate genes [10]. We selected a panel of genes that are known to cause congenital skeletal muscle disorders and looked for their expression in Sol8 Gne^KO^ cells compared to wild-type cells (Figure 4A). The heatmap shows the distinct transcriptomic landscape between wild-type myoblasts and myotubes as well as impaired expression of many genes in the Gne^KO^ clone. Using the STRING database for visualization, it became clear that most of the altered gene products form a tight network (Figure 4B). Among the most interesting candidates (highlighted in red) were the sodium channel protein type 4 subunit α (Scn4a), ryanodine receptor 1 (Ryr1), and glycogen phosphorylase muscle form (Pygm). These are known to play important roles in excitation-contraction coupling and energy metabolism, respectively. Diminished expression of these three genes was validated in Sol8 cells via qPCR (Figure 4C–E).

### 3.5. Myopathogene Expression in the C2C12 Gne^KO^ Model

**Figure 5:**
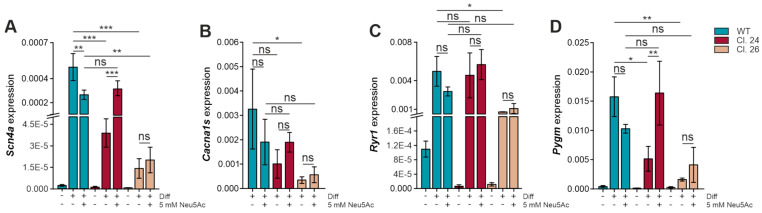
Expression analysis of muscle-specific genes in C2C12 wild-type and Gne^KO^ cells. qPCR analysis was used to examine gene expression in C2C12 wild-type (WT) and Gne^KO^ (Cl. 24 and Cl. 26) myoblasts and myotubes. Where indicated, differentiation medium was supplemented with 5 mM Neu5Ac for the duration of the entire differentiation protocol. (**A**) mRNA expression of the sodium channel protein type 4 subunit α (Scn4a). (**B**) mRNA expression of voltage-dependent L-type calcium channel subunit alpha-1S (Cacna1s). (**C**) mRNA expression of the ryanodine receptor 1 (Ryr1). (**D**) mRNA expression of the muscle-specific glycogen phosphorylase (Pygm). All values represent the ΔCt value norm. to Gapdh expression. Diff: differentiated, Neu5Ac: *N*-Acetylneuraminic acid. All graphs represent the mean of three independent experiments ± standard deviation. Asterisks indicate the *p*-value of the sample compared to the respective condition. Statistical analysis: One-way ANOVA. ns = non-significant, * *p* < 0.05, ** *p* < 0.01, *** *p* < 0.001.

To confirm that down-regulation of Scn4a, Ryr1, and Pygm is directly linked to the lack of Gne expression, we also checked expression levels of these genes in the C2C12 Gne^KO^ model and expanded the panel to include the voltage-dependent L-type calcium channel subunit α-1S (Cacna1s). Similarly to Sol8 cells, all genes were hardly expressed in myoblasts, but expression increased significantly with differentiation (Figure 5A,C,D). Scn4a, Cacna1s, and Pygm had lower expression in C2C12 Gne^KO^ myotubes compared to wild-type control cells, even though Cacna1s levels in clone #24 were statistically not significant (*p* = 0.05546) (Figure 5A,B,D). Note that Ryr1 expression in clone #24 was comparable to wild-type cells, while it was reduced in clone #26 (Figure 5C). Interestingly, Scn4a, Cacna1s, and Pygm are skeletal muscle tissue-specific proteins, while Ryr1 is also found in brain tissue and, to a lesser extent, in heart tissue [22,23,24]. Thus, aberrant Ryr1 expression might not be a specific consequence of Gne depletion but could also be due to single-cell heterogeneity and needs further investigation.

We previously observed that exogenous *N*-acetylneuraminic acid (Neu5Ac) supplementation during the differentiation process had a positive effect on sialylation and myotube formation in C2C12 Gne^KO^ cells, while it negatively impacted wild-type differentiation [11]. Therefore, the next step was to evaluate the effects of Neu5Ac supplementation on gene expression to see whether impaired gene expression can be rescued by artificial sialylation of the cells. Noteworthy, excess Neu5Ac tended to negatively affect Scn4a, Cacna1s, Ryr1, and Pygm expression in wild-type myotubes. However, Gne^KO^ clone #24 showed a positive response to Neu5Ac, while the effects were less pronounced in clone #26 (Figure 5).

### 3.6. Disturbed Energy Metabolism in C2C12 Gne^KO^ Cells

**Figure 6:**
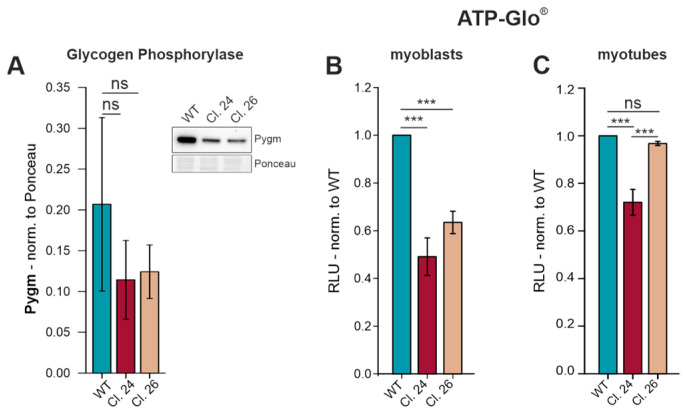
Reduced glycogen phosphorylase protein expression and decreased ATP production in C2C12 Gne^KO^ cells. (**A**) Western blot analysis of muscle-specific glycogen phosphorylase (Pygm) protein expression in differentiated C2C12 wild-type (WT) and Gne^KO^ clones #24 and #26. ATP-Glo^®^ assay for the quantitative analysis of intracellular ATP levels in C2C12 wild-type (WT) and Gne^KO^ (Cl. 24 and Cl. 26) (**B**) myoblasts and (**C**) differentiated myotubes, normalized to wild-type cells. RLU: relative luminescent units. Bar graphs show the mean of three independent experiments ± standard deviation. Asterisks indicate the *p*-value of the sample compared to the respective condition. Statistical analysis: One-way ANOVA, ns = non-significant, *** *p* < 0.001.

Skeletal muscle tissue has a high energy demand, mainly because of the cross-bridge cycle of sarcomere contraction. Glycogen is the major glucose storage form in myofibers, and glycogen phosphorylase is needed for glycogen breakdown into glucose-1-phosphate to make it accessible for metabolism and subsequent ATP generation. Along with reduced mRNA levels of Pygm in C2C12 Gne^KO^ myotubes (Figure 5), protein expression was similarly affected (Figure 6A). To assess whether reduced Pygm expression has direct effects on energy metabolism, intracellular ATP levels were quantified using the ATP-Glo^®^ assay. Gne^KO^ myoblasts contained 40–50% less ATP than wild-type myoblasts (Figure 6B). Intriguingly, only Gne^KO^ clone #24 myotubes exhibited a reduction in ATP, while clone #26 had comparable amounts as wild-type myotubes (Figure 6C).

### 3.7. Voltage-Dependent L-Type Calcium Channel Subunits Are Differentially Expressed in C2C12 Gne^KO^ Cells

**Figure 7:**
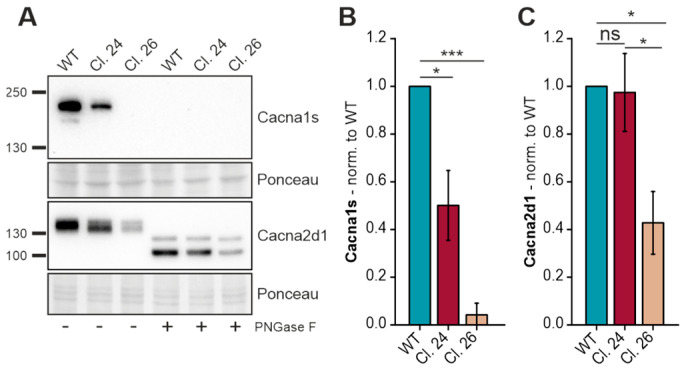
Reduced expression of two voltage-dependent L-type calcium channel subunits in C2C12 Gne^KO^ myotubes. (**A**) Western blot showing the protein expression of the voltage-dependent L-type calcium channel subunits α-1s (Cacna1s) and α-2/δ-1 (Cacna2d1). (**B**) Quantitative analysis of Cacna1s protein expression normalized to ponceau and wild-type (WT) expression. (**C**) Quantitative analysis of Cacna2d1 protein expression normalized to ponceau and wild-type (WT) expression. Bar graphs show the mean of three independent experiments ± standard deviation. Asterisks indicate the *p*-value of the sample compared to the respective condition. Statistical analysis: One-way ANOVA. ns = non-significant, * *p* < 0.05, *** *p* < 0.001.

A direct link between the sialylation status and channel function of Scn4a was already described [25]. However, not much is known about sialylation and the calcium channel CaV1.1, of which Cacna1s is the pore-forming subunit. Therefore, we examined Cacna1s and Cacna2d1 protein expression, as both subunits have potential N-glycosylation sites. Protein expression of Cacna1s mirrored the qPCR data, as there was a strong decline in protein expression from wild-type myotubes to Gne^KO^ clone #24, to no protein detection in clone #26 (Figure 7A,B). Interestingly, upon PNGase F digestion of the protein lysates, the Cacna1s signal disappeared. As PNGase F is a peptide:N-glycosidase that efficiently removes N-glycans from the protein backbone, we speculate that the antibody used to detect Cacna1s recognizes the glycosylated form only. Cacna2d1 regulates the activation/inactivation kinetics of the calcium channel, and like Cacna1s, Cacna2d1 expression was lowest in Gne^KO^ clone #26 (Figure 7A,C). Even though the signal intensity in Gne^KO^ clone #24 was similar to wild-type myotubes, there was a slight shift in the molecular weight detectable in clone #24 and clone #26, most likely due to missing sialic acids. PNGase F digestion showed a clear size shift compared to the glycosylated protein, providing evidence for the existence of N-glycans on Cacna2d1 (Figure 7A).

## 4. Discussion

GNE myopathy is a rare neuromuscular disorder that solely affects skeletal muscle tissue. First symptoms usually appear during early adulthood; hence, embryonic and early development are not impaired in the patients. The results generated in this study showed that muscle cells that lack Gne protein expression not only present hyposialylation but also alterations in other glycosylation pathways. Profiling of the N-glycans of C2C12 wild-type and Gne^KO^ myoblasts confirmed the reduction in sialoglycans with a concomitant increase in Gal-α-Gal epitopes. Interestingly, a strong increase in N-glycan branching (number of antennae) was observed in Gne^KO^ myoblasts, which was described before for GNE-silenced cells [26]. However, PHA-L lectin binding is inhibited by terminal α-2,6-sialic acids; hence, it could be an indirect effect of missing sialic acids [27]. These results show that even minor alterations in glycan structures might have major effects on ligand binding and intercellular communication. Skeletal muscle tissue has the capability to regenerate after injury, a process that relies on the proper function of muscle stem cells (satellite cells). The C2C12 myoblast cell line mimics the satellite cells to some extent, as it can be differentiated into multi-nucleated myotubes. Intriguingly, Gne knockout impairs this differentiation process, leading to the formation of myotubes with abnormal morphology. Satellite cell and myoblast activation depends on external signals coming from the extracellular milieu; thus, one could speculate that alterations in the myoblast glycome could lead to aberrant signaling and impaired differentiation. In fact, skeletal muscle tissue of GNEM patients is replaced by fat and connective tissue, leading to muscle atrophy. The characteristic alterations in the extracellular matrix of C2C12 Gne^KO^ clone #24 myotubes should be subject to further investigations, as they could give insights into possible fibrotic mechanisms.

Mutations in hundreds of different genes are linked to congenital myopathies, and they are collectively referred to as myopathogenes. Transcriptomic analysis revealed that many of those myopathogenes are dysregulated in Gne^KO^ myotubes. Computational interaction networks suggest a high interdependency between the gene products, rendering the physiological interactions susceptible to damage even upon slight alterations of gene expression. Interestingly, electromyography (EMG) patterns of GNEM subjects indicate more or less abundant spontaneous potentials of the examined muscles [28]. One of the strongly down-regulated genes was Scn4a, the α subunit of the skeletal muscle-specific voltage-dependent sodium channel Nav1.4. Scn4a was down-regulated in Sol8 Gne^KO^ cells and C2C12 Gne^KO^ clones, as well as in the transcriptomic data of a patient biopsy sample of affected tissue, hinting towards a Gne-specific effect [29]. As sialylation was found to be important for NaV1.4 function, two explanations are possible. First, missing sialic acids lead to the rapid degradation of Scn4a and to diminished gene expression to prevent the incorporation of a malfunctioning sodium channel into the sarcolemma. Alternatively, the high degree of Scn4a sialylation was proposed to add to the maintenance of the negative membrane potential, keeping the cells in a resting state [30]. Thus, upon impaired GNE function, hyposialylation could cause slight alterations in the membrane potential, shifting the resting potential to a less negative value, triggering spontaneous potentials in the EMG profiles of GNEM patients. Diminished expression of the downstream effectors Cacna1s, Cacna2d1, and Ryr1 could be due to low abundance or dysfunction of Scn4a. Together, these perturbations in gene and protein expression could impede efficient excitation-contraction coupling. Contraction of the sarcomeres is an energy-consuming process that requires ATP. Recently, glycogen accumulation in the biopsies of GNEM patients was reported [31]. Our C2C12 Gne^KO^ cell model, as well as the Sol8 Gne^KO^ cells, showed reduced expression of Pygm, suggesting impaired degradation of glycogen and energy metabolism. This might also be a secondary effect in response to impaired sarcomere contraction and thus lower energy demands of the cells.

**Figure 8:**
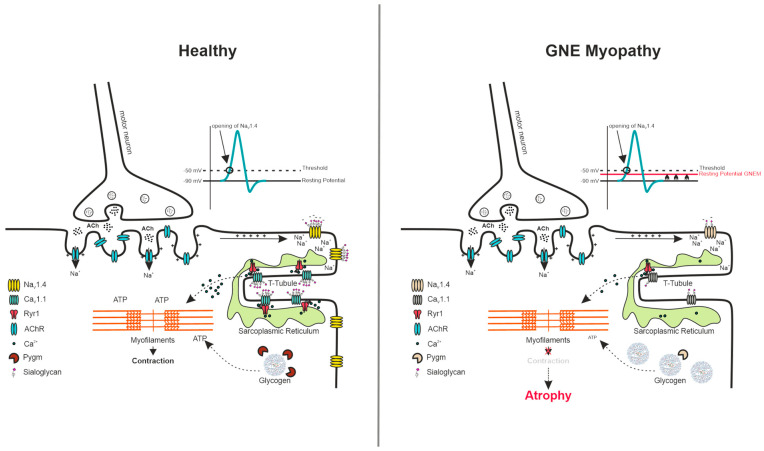
Possible disease mechanism of GNE myopathy. Alterations in the membrane potential of myofibers, due to hyposialylation, could cause an atrophic signaling cascade. Acetylcholine receptors at the neuromuscular junction receive the action potential from the motor neurons and pass the signal to voltage-gated sodium channel NaV1.4. The NaV1.4 opening leads to depolarization and conformational changes in CaV1.1 that in turn activate the ryanodine receptor in the sarcoplasmic reticulum, resulting in Ca^2+^ efflux. See main text for further description. NaV1.4: voltage-gated sodium channel 1.4, CaV1.1: voltage-gated calcium channel 1.1, Ryr1: ryanodine receptor 1, ACh: acetylcholine, AChR: acetylcholine receptor, Pygm: muscle-specific glycogen phosphorylase, ATP: adenosine triphosphate.

In conclusion, hyposialylation of C2C12 myoblasts causes alterations in the glycomic landscape of these cells. These changes could be the cause of abnormal differentiation and reduced expression of certain myopathogenes. Missing sialic acids could also have detrimental effects on certain protein functions, like the sodium and calcium channels that are needed for propagation of the action potential and downstream excitation-contraction coupling. Figure 8 depicts the possible mechanisms that could contribute to the disease manifestation of GNE myopathy, based on the results from our C2C12 Gne^KO^ cell model. Interestingly, an excess of sialic acids had a negative effect on C2C12 wild-type cell physiology, which should be kept in mind in regard to supplementation as a putative therapeutic strategy. This study adds new aspects to how hyposialylation could lead to the manifestation of GNE myopathy in young adults and might open new routes for therapeutic interventions. The limitations we want to address are that the C2C12 and Sol8 Gne knockout cell lines represent a model for basic research only. Both cell lines are mouse-derived, immortalized cell lines, and the findings should thus be interpreted with care. However, as RNAseq data from patient-derived samples support our findings, these results could contribute to more translational experiments using patient-derived stem cells. As GNE myopathy is a rare disease, patient material is not easily available, and thus, basic models could support the research efforts in the search for suitable therapies in the future.

## Figures and Tables

**Table 1:** Antibodies and staining reagents.

Antibody/Lectin	Host Species	Dilution	Company
Myh (Western blot)	mouse	1:1000	Santa Cruz Biotechnology (sc-376157)
Myh (IF)	mouse	1:100	Santa Cruz Biotechnology (sc-376157)
O-GlcNAc (CTD110.6)	mouse	1:1000	Cell Signaling Technology (#9875)
Pygm (OTI5D1)	rabbit	1:1000	Invitrogen (MA5-27442)
Cacna1s [1A]	mouse	1:1000	Abcam (ab2862)
Cacna2d1 [EPR23267-8]	rabbit	1:1000	Abcam (ab253190)
Hoechst H33258		1:1000	Sigma-Aldrich (#94403)
Lectin PHA-L, Alexa Fluor 488			Thermo Fisher (L11270)

**Table 2:** Primer Sequences for qPCR.

Name	Sequence (5′–3′)
*Gapdh fwd*	CCTGGAGAAACCTGCCAAGTATG
*Gapdh rev*	AGAGTGGGAGTTGCTGTTGAAGTC
*Scn4a fwd*	TTCTCGGAGCCTGAGGACATCA
*Scn4a rev*	GTGAAACACTCCTCAGGTAGCTC
*Pygm fwd*	CCCTACCCACTTTGGAACCC
*Pygm rev*	GTGCACTTGGTTAGACCCCA
*Ryr1 fwd*	TTTCCTGGACCGAGTGTATGGC
*Ryr1 rev*	CAGACAGAGGTAGCGGTTCAGT
*Cacna1s fwd*	CCTGGCTATTGCTGTGGACAAC
*Cacna1s rev*	CTGCTCCAGTTTCTTGGTCACC

## Data Availability

The data presented in this study are available in Gene Expression Omnibus—NCBI—NIH at https://doi.org/10.3389/fgeed.2022.930110, reference number GSE202046.

## Abbreviations

The following abbreviations are used in this manuscript:

GNE	UDP-*N*-acetylglucosamine 2-epimerase/*N*-acetylmannosamine kinase
GNEM	GNE Myopathy
GlcNAc	*N*-acetylglucosamine
ManNAc	*N*-acetylmannosamine
Neu5Ac	*N*-acetylneuraminic acid
WT	Wild-type
KO	Knockout
GalNAc	*N*-acetylgalactosamine
